# Primary Progressive Multiple Sclerosis With Acute Neurologic Deterioration Secondary to Metabolic Derangements and Medication Effects

**DOI:** 10.7759/cureus.107178

**Published:** 2026-04-16

**Authors:** Vivek Boddakayala, Amir Mirnateghi, Konstantin M Nakov, Mithun Pattathan

**Affiliations:** 1 Medicine, Lake Erie College of Osteopathic Medicine, Bradenton, USA; 2 Internal Medicine, St. Vincent's Medical Center, Jacksonville, USA

**Keywords:** acute kidney injury, hypercalcemia, neurodegenerative disease, primary progressive multiple sclerosis, pseudoexacerbation

## Abstract

A 73-year-old man with a 33-year history of primary progressive multiple sclerosis (PPMS) presented with subacute gait instability, confusion, dysarthria, and severe muscle spasms. Laboratory evaluation revealed severe acute kidney injury and hypercalcemia, while noncontrast brain MRI demonstrated chronic periventricular demyelinating changes without new inflammatory lesions; contrast-enhanced MRI was not obtained. The clinical picture was most consistent with a metabolically driven pseudoexacerbation precipitated by hypercalcemia from prolonged immobilization, nephrotoxic polypharmacy, and subtherapeutic gabapentin dosing, though concurrent inflammatory PPMS activity could not be excluded. Treatment with high-dose intravenous methylprednisolone, intravenous fluid resuscitation, and calcitonin resulted in normalization of renal function and serum calcium and meaningful improvement in neurologic symptoms. This case underscores the importance of thorough systemic evaluation before attributing acute neurologic deterioration in PPMS to inflammatory relapse.

## Introduction

Multiple sclerosis (MS) is a chronic autoimmune demyelinating disease of the central nervous system characterized by inflammation, demyelination, gliosis, and axonal loss, resulting in varied and progressive neurologic dysfunction [1]. MS is classified into distinct clinical subtypes that differ in their disease course, inflammatory burden, and responsiveness to therapy. Relapsing-remitting MS (RRMS) accounts for approximately 85% of initial diagnoses and is characterized by discrete episodes of neurologic dysfunction (relapses) lasting at least 24 hours and followed by partial or complete recovery [2]. Primary progressive MS (PPMS), by contrast, affects approximately 10%-15% of MS patients and is defined by continuous neurologic deterioration from disease onset without distinct relapses or remissions. PPMS typically demonstrates less gadolinium-enhancing lesion activity on MRI than RRMS, tends to be less responsive to corticosteroids and conventional disease-modifying therapies (DMTs), and carries a greater burden of early disability [2]. Ocrelizumab, an anti-CD20 monoclonal antibody, remains one of the few DMTs with demonstrated, albeit modest, efficacy in slowing disability progression in PPMS.

A true MS exacerbation reflects new inflammatory activity in the central nervous system, producing new or worsening neurologic symptoms lasting at least 24 hours in the absence of a systemic cause [3]. In contrast, a pseudoexacerbation refers to a temporary worsening of preexisting MS symptoms precipitated by a reversible systemic trigger (such as infection, fever, metabolic imbalance, or medication effects) without new central nervous system inflammation [4]. The clinical distinction between these two entities has significant therapeutic implications: true relapses may warrant immunomodulatory therapy, while pseudoexacerbations require identification and correction of the underlying systemic trigger. Importantly, pseudoexacerbations may coexist with or be superimposed on genuine inflammatory activity, creating a complex, overlapping clinical picture that demands thorough evaluation [5].

In this report, we describe a patient with longstanding PPMS whose acute neurologic deterioration was significantly influenced by a combination of severe hypercalcemia, acute kidney injury (AKI), and medication-related factors. Several features distinguish this case and justify its presentation. First, the combination of three concurrent metabolic contributors (hypercalcemia from prolonged immobilization, AKI from nephrotoxic polypharmacy, and subtherapeutic gabapentin dosing due to nonadherence) created a uniquely complex pseudoexacerbation syndrome that mimics inflammatory relapse. Second, without contrast MRI, active demyelination could not be ruled out, highlighting the diagnostic uncertainty that arises when imaging is incomplete. Third, despite PPMS's characteristically limited responsiveness to corticosteroids, the patient showed meaningful neurologic improvement when metabolic correction and empiric steroid therapy were pursued concurrently. This raised questions about the overlap between systemic and inflammatory contributors in this population. This case highlights the critical importance of comprehensive systemic evaluation before attributing acute neurologic decline in PPMS to inflammatory disease activity.

## Case presentation

Patient information

The patient is a 73-year-old man (height 185 cm, weight 78.8 kg, BMI 23.0 kg/m²) with a 33-year history of PPMS. His documented comorbidities include chronic cholecystitis. Documented allergies include latex, penicillin, and statins. He reported no history of tobacco or alcohol use.

History of present illness

Approximately eight weeks before admission, the patient's family noted progressive deterioration in his gait and balance. He transitioned from using a cane to requiring a walker during this period. Before this acute decline, he had maintained relative functional stability, ambulating independently with a cane and managing most activities of daily living with minimal assistance, corresponding to an estimated Expanded Disability Status Scale (EDSS) score of approximately 4.0-5.0. This reflects moderate disability with full ambulatory capacity. He had not received neurologic follow-up for approximately six years before this admission, and his only prior DMT was interferon beta-1b (Betaseron), which had been discontinued due to intolerance. No alternative DMT was initiated after discontinuation.

Seven days prior to admission, he developed marked bilateral lower extremity weakness, reduced mobility, poor oral intake, and progressive dysphagia. He had a longstanding history of dysphagia and chronic cough, both likely attributable to his underlying MS. Three days before presentation, he developed severe and episodic muscle spasms involving the extremities. His partner noted increasing confusion, significant dysarthria, and nonadherent subtherapeutic gabapentin use; the patient had been taking one tablet daily rather than the prescribed regimen of six.

Three weeks before admission, the patient developed low-grade fever and dark urine. Given his mobility limitations, he had been using a urinal bottle and had limited access to urologic evaluation. His primary care physician treated a presumed urinary tract infection with a 10-day course of trimethoprim-sulfamethoxazole (TMP-SMX), which was completed one day before hospital presentation. He was brought to the emergency department following a severe spasm episode, at which time laboratory studies and imaging were obtained.

Laboratory and imaging findings

A comprehensive metabolic panel obtained on arrival to the emergency department revealed severe AKI and hypercalcemia (Table 1). Serum creatinine was markedly elevated at 4.12 mg/dL (reference 0.73-1.18 mg/dL), blood urea nitrogen (BUN) was 65 mg/dL (reference 7-25 mg/dL), and estimated glomerular filtration rate (GFR) was critically reduced at 15 mL/minute/1.73 m², consistent with stage 4 AKI [6]. Serum calcium was severely elevated at 14.5 mg/dL (reference 8.5-10.5 mg/dL). Sodium was at the upper limit of normal at 145 mmol/L, consistent with volume depletion contributing to both the AKI and hypercalcemia. Parathyroid hormone (PTH), parathyroid hormone-related peptide (PTHrP), and vitamin D metabolite levels were not obtained during this admission, representing a limitation in fully characterizing the etiology of hypercalcemia, as discussed below.

**Table 1:** Admission and discharge laboratory findings Discharge values reflect normalization following intravenous fluid resuscitation, calcitonin administration, and discontinuation of nephrotoxic agents BUN: blood urea nitrogen; AKI: acute kidney injury; eGFR: estimated glomerular filtration rate

Parameter	Patient value	Reference range	Clinical significance
BUN	65 mg/dL	7-25 mg/dL	Elevated AKI
Creatinine	4.12 mg/dL	0.73-1.18 mg/dL	Elevated AKI
eGFR (estimated)	15 mL/minute/1.73 m²	>60 mL/minute/1.73 m²	Severely reduced
Calcium	14.5 mg/dL	8.5-10.5 mg/dL	Severe hypercalcemia
Sodium	145 mmol/L	135-145 mmol/L	Normal
Potassium	4.0 mmol/L	3.5-5.4 mmol/L	Normal
Chloride	111 mmol/L	98-110 mmol/L	Mildly elevated
Glucose	110 mg/dL	70-100 mg/dL	Mildly elevated
BUN at discharge	27 mg/dL	7-25 mg/dL	Near normal
Creatinine at discharge	1.14 mg/dL	0.73-1.18 mg/dL	Normal
Calcium at discharge	8.2 mg/dL	8.5-10.5 mg/dL	Normal

Imaging

**Figure 1:**
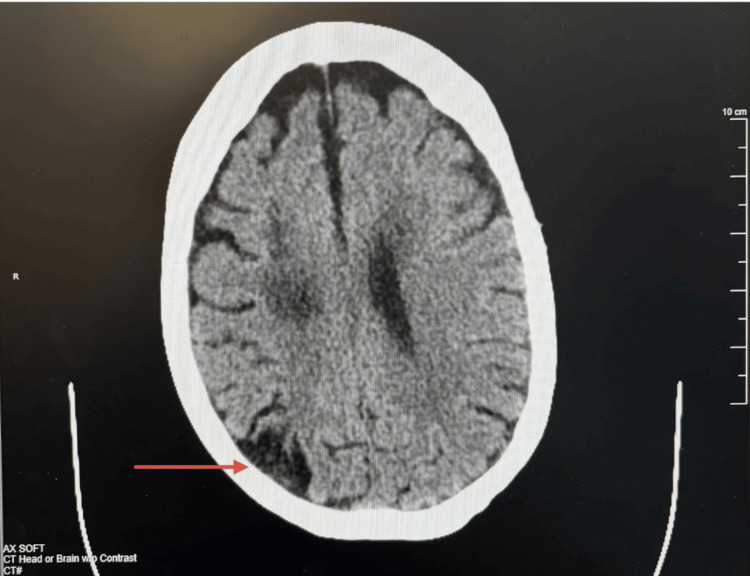
CT of the head/brain (noncontrast) showing chronic right parieto-occipital infarct and probable chronic basal ganglia and left thalamic infarcts (red arrow displays right parieto-occipital infarct)

**Figure 2:**
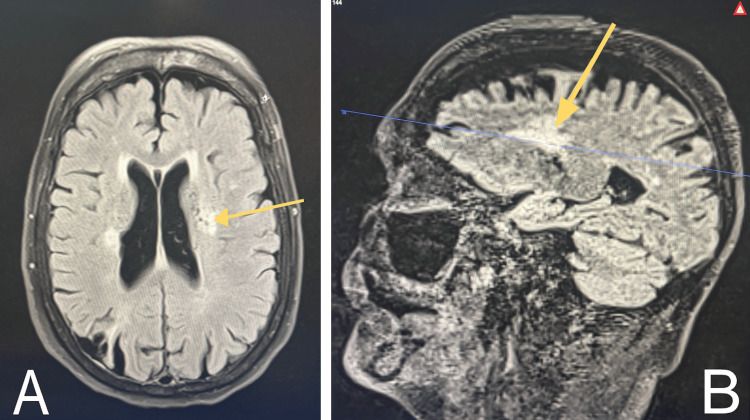
Noncontrast MRI of the brain showing extensive periventricular T2 FLAIR hyperintensities consistent with chronic demyelination (yellow arrows display hyperintensities). (A) Axial view. (B) Sagittal view FLAIR: fluid-attenuated inversion recovery

Noncontrast CT of the head demonstrated no acute intracranial abnormalities. Chronic findings included moderate white matter microangiopathic changes, a chronic right parietooccipital infarct, and probable chronic basal ganglia and left thalamic infarcts (Figure 1). Noncontrast MRI of the brain subsequently demonstrated extensive periventricular T2/fluid-attenuated inversion recovery (FLAIR) hyperintensities consistent with chronic demyelination (Figure 2). No gadolinium-enhancing lesions were identified, as contrast was not administered during this admission. Spinal cord MRI was not obtained. The absence of contrast MRI represents a significant limitation, as it limits the ability to assess blood-brain barrier disruption or active inflammation. The imaging findings were therefore interpreted as consistent with chronic, inactive PPMS, though active demyelination could not be fully excluded.

Neurological examination

A detailed neurological examination was performed on admission and is summarized in Table 2.

**Table 2:** Admission neurological examination findings RUE: right upper extremity; LUE: left upper extremity; RLE: right lower extremity; LLE: left lower extremity; DTR: deep tendon reflex; CN: cranial nerve; UMN: upper motor neuron; PPMS: primary progressive multiple sclerosis

Domain	Finding	Clinical interpretation
Mental status	Alert, oriented ×3; intact attention, comprehension, and executive function	No encephalopathy; cognitive function preserved despite metabolic disturbances
Language	Dysarthria present; no aphasia	Consistent with lower motor neuron facial weakness (CN VII); language pathways intact
CN II	Visual acuity 20/30 bilaterally; intact visual fields and pupillary light reflex	No optic neuritis; visual pathway intact
CN III/IV/VI	Intact extraocular movements; no ptosis or nystagmus	No internuclear ophthalmoplegia
CN V	Decreased sensation left hemiface; normal jaw strength, no deviation	Trigeminal sensory involvement consistent with demyelinating disease
CN VII	Left lower facial droop; UMN pattern (forehead sparing)	Central facial palsy consistent with a contralateral corticobulbar tract lesion
CN VIII	Normal hearing bilaterally	No vestibulocochlear involvement
CN IX/X	Normal palatal elevation; dysphagia present	Dysphagia likely multifactorial: corticobulbar dysfunction and prolonged immobility
CN XI	Decreased left trapezius strength	Left accessory nerve involvement
CN XII	No tongue deviation or protrusion abnormality	Hypoglossal nerve intact
Motor tone	Normal tone in all four limbs	No spasticity at the time of examination
Motor strength	RUE 5/5, LUE 4/5, RLE 4/5, LLE 4/5	Left-predominant weakness; right lower extremity also reduced, consistent with PPMS burden
Sensory	Intact light touch, pinprick, vibration, and proprioception	No sensory level; peripheral sensory pathways intact
Coordination	No dysmetria on finger-nose-finger or heel-shin	Cerebellar pathways intact
Reflexes	DTRs 2+ throughout; negative Babinski bilaterally; no clonus	No upper motor neuron release signs at the time of examination

Initial diagnostic assessment

On initial assessment, the patient was diagnosed with a suspected multiple sclerosis exacerbation in the setting of concurrent AKI and severe hypercalcemia, both likely multifactorial in origin. Dehydration, prolonged immobilization, and medication-related nephrotoxicity were considered the primary contributors to the renal and metabolic derangements. Given the absence of gadolinium-enhancing lesions on MRI and the presence of significant systemic stressors, the possibility of a pseudoexacerbation or a pseudoexacerbation superimposed on underlying PPMS progression was carefully considered and discussed with consulting services.

Hospital course and management

Management addressed the neurologic, renal, and metabolic dimensions of the patient's presentation concurrently. For the presumed MS component, the patient received high-dose intravenous methylprednisolone in accordance with standard acute relapse management, despite his documented history of an adverse psychiatric response to corticosteroids, which had previously manifested as delirious ideation and hyperactivity, given the severity of his neurologic presentation. Close psychiatric monitoring was maintained throughout steroid administration. Supportive neurologic care included swallowing therapy with a modified liquid diet for dysphagia, wound care for sacral pressure injuries, and physical therapy to address mobility deficits.

Nephrology was consulted and attributed the AKI to a combination of volume depletion and nephrotoxic polypharmacy, specifically the recent use of TMP-SMX and losartan. Both agents were discontinued on admission. Gabapentin was held given the risk of accumulation in the setting of severely impaired renal clearance and was restarted at an appropriate dose once renal function recovered. Aggressive intravenous fluid resuscitation with lactated Ringer's solution was initiated to support renal perfusion and promote volume expansion.

Endocrinology was consulted for the severe hypercalcemia. The thyroid ultrasound was normal. In the absence of PTH or vitamin D levels, the hypercalcemia was attributed most likely to immobilization-induced osteoclast activation in the context of significant dehydration, though primary hyperparathyroidism and other etiologies could not be formally excluded. Vitamin D supplementation was withheld. Calcitonin was administered to acutely lower serum calcium levels, with concurrent volume expansion through intravenous fluids; serial calcium measurements were obtained until normalization.

Tele-neurology was consulted and confirmed the prior diagnosis of PPMS in the absence of new inflammatory MRI lesions, recommending continuation of IV methylprednisolone and outlining a plan for formal outpatient neurologic follow-up to evaluate eligibility for ocrelizumab. By discharge, renal function and serum calcium had normalized (BUN 27 mg/dL, creatinine 1.14 mg/dL, calcium 8.2 mg/dL). The patient demonstrated meaningful clinical improvement in dysarthria, muscle strength, and spasticity, though a formal posttreatment EDSS was not recorded. He was transferred to an inpatient rehabilitation facility with neurology follow-up arranged.

## Discussion

This case illustrates the diagnostic and therapeutic complexity that arises when acute neurologic deterioration in a patient with PPMS is precipitated or amplified by concurrent systemic and metabolic disturbances. The central clinical question, whether this patient experienced a true inflammatory MS relapse, a pseudoexacerbation driven by metabolic derangements, or a combination of both, could not be definitively resolved given the absence of gadolinium-enhanced MRI. However, several lines of evidence favor a predominant pseudoexacerbation mechanism with possible underlying PPMS progression.

Pseudoexacerbations are well-recognized in MS and are defined as transient worsening of established neurologic deficits triggered by reversible systemic factors, without new central nervous system inflammatory activity [4]. Common triggers include infection, fever, metabolic derangements, and medication effects [5]. This patient was simultaneously exposed to at least three significant neurologic stressors: severe hypercalcemia (calcium 14.5 mg/dL), stage 4 AKI (creatinine 4.12 mg/dL, eGFR 15), and subtherapeutic gabapentin dosing due to nonadherence. The convergence of these factors in a patient with longstanding PPMS created an environment conducive to symptomatic deterioration independent of new demyelination.

Severe hypercalcemia exerts direct neurologic effects through multiple mechanisms. Elevated extracellular calcium stabilizes neuronal membrane resting potential, raising the threshold for action potential generation and impairing neuromuscular excitability [7]. At calcium levels exceeding 14 mg/dL, patients may develop confusion, dysarthria, proximal muscle weakness, and profound fatigue, all features prominently present in this patient. In the setting of preexisting demyelination, the threshold at which hypercalcemia produces clinically apparent neurologic dysfunction is likely substantially lower than in neurologically intact individuals. Concurrent AKI further compounded neurologic dysfunction through impaired clearance of gabapentin and other renally excreted metabolites, and through the general proencephalopathic nature of uremia [8]. The marked clinical improvement following metabolic correction (normalization of calcium, creatinine, and BUN) lends strong support to the hypothesis that these derangements were primary drivers of the acute deterioration.

The attribution of hypercalcemia to immobilization-induced bone resorption is clinically plausible given the patient's prolonged reduced mobility and significant dehydration. Immobilization hypercalcemia results from uncoupled osteoclast-mediated bone resorption in the absence of weight-bearing, and it is well recognized in chronically immobile patients [7]. However, the absence of PTH, PTHrP, and vitamin D metabolite levels represents a limitation of this evaluation. Primary hyperparathyroidism, granulomatous disease, and occult malignancy cannot be formally excluded without these studies. Future management of this patient should include a complete hypercalcemia workup.

Regarding the role of TMP-SMX, this antibiotic contributes to AKI through multiple mechanisms, including tubular toxicity, crystal nephropathy, and, via its trimethoprim component, inhibition of tubular creatinine secretion, which can falsely elevate measured creatinine without reflecting true GFR decline [8]. The clinical picture is further complicated by concurrent losartan use, which reduces glomerular filtration pressure through efferent arteriolar dilation and exacerbates volume depletion-related AKI. Together, these agents in the setting of reduced oral intake and dehydration likely produced the severe renal impairment observed on admission.

With respect to the MS component, the imaging findings argue against a dominant inflammatory relapse. Noncontrast brain MRI demonstrated exclusively chronic T2/FLAIR periventricular hyperintensities without features suggesting acute demyelination, such as new T2 lesions, lesion enlargement, or critical gadolinium enhancement indicating blood-brain barrier disruption. In PPMS, gadolinium-enhancing lesions are inherently less frequent than in RRMS; however, their presence would have strongly supported concurrent inflammatory activity. Their absence, though not conclusive given that contrast was not administered, combined with the resolution of symptoms following metabolic correction, makes a dominant pseudoexacerbation the most likely explanation. However, corticosteroids may have contributed to neurologic improvement through anti-inflammatory mechanisms even in PPMS, and the possibility that both processes were simultaneously active cannot be excluded [3].

From a clinical decision-making standpoint, this case underscores the importance of a systematic approach to acute neurologic worsening in PPMS. Before attributing deterioration to inflammatory relapse, clinicians should actively screen for reversible systemic contributors, including infection, electrolyte derangements, renal and hepatic dysfunction, medication toxicity, and heat exposure, given that treating a pseudoexacerbation with immunosuppression alone, without addressing the underlying trigger, is unlikely to produce meaningful improvement. When metabolic and neurologic contributors coexist, as in this patient, concurrent management of both is appropriate. The adverse psychiatric response to prior steroid exposure (delirious ideation and hyperactivity) also highlights the importance of careful risk-benefit assessment and close monitoring when corticosteroids are used in patients with a documented intolerance history.

## Conclusions

This case describes a 73-year-old man with longstanding PPMS whose acute neurologic deterioration was most consistent with a metabolically driven pseudoexacerbation, substantially precipitated by severe hypercalcemia, stage 4 AKI, and subtherapeutic gabapentin dosing due to nonadherence, in the context of prolonged immobility, dehydration, and nephrotoxic polypharmacy. While concurrent underlying PPMS progression cannot be excluded, particularly given the absence of gadolinium-enhanced and spinal cord MRI, the marked improvement in neurologic symptoms following metabolic correction supports systemic derangements as the predominant contributor to the acute presentation. Empiric corticosteroid therapy was administered concurrently and may have provided additional benefit, though the contribution of inflammatory disease activity remains uncertain.

This case reinforces three key clinical lessons. First, acute neurologic worsening in PPMS should prompt thorough systemic evaluation before a diagnosis of inflammatory relapse is assumed, as reversible metabolic contributors may represent the primary or sole driver of deterioration. Second, a complete imaging evaluation, including gadolinium-enhanced MRI and spinal cord imaging, is essential to distinguish active demyelination from pseudoexacerbation; the absence of contrast-enhanced MRI in this case represents a significant diagnostic limitation. Third, patients with PPMS who have lapsed from neurologic care are at elevated risk for compounding vulnerabilities, medication nonadherence, unmanaged systemic comorbidities, and delayed access to DMT, all of which contributed to this patient's presentation. Maintaining neurologic follow-up and addressing systemic health in PPMS are therefore critical components of long-term disease management.
